# The potential health and revenue effects of a tax on sugar sweetened beverages in Zambia

**DOI:** 10.1136/bmjgh-2019-001968

**Published:** 2020-04-29

**Authors:** Peter Hangoma, Maio Bulawayo, Mwimba Chewe, Nicholas Stacey, Laura Downey, Kalipso Chalkidou, Karen Hofman, Mpuma Kamanga, Anita Kaluba, Gavin Surgey

**Affiliations:** 1 Department of Health Policy and Management, University of Zambia, Lusaka, Zambia; 2 SA MRC/ Wits Centre for Health Economics and Decision Science, University of Witwatersrand School of Public Health, Faculty of Health Sciences, Johannesburg, South Africa; 3 School of Public Health, Imperial College London, London, UK; 4 Institute of Global Health Innovation, Imperial College London, London, United Kingdom; 5 Center for Global Development, Washington, DC, USA; 6 Ministry of Health, Lusaka, Zambia; 7 Health Economics and HIV and AIDS Research Division (HEARD), University of KwaZulu-Natal, Durban, South Africa

**Keywords:** health policy, health economics, public health

## Abstract

The global burden of non-communicable diseases (NCDs) has been rising. A key risk factor for NCDs is obesity, which has been partly linked to consumption of sugar sweetened beverages (SSBs). A tax on SSBs is an attractive control measure to curb the rising trend in NCDs, as it has the potential to reduce consumption of SSBs. However, studies on the potential effects of SSB taxes have been concentrated in high-income countries with limited studies in low-income and middle-income countries. Using data from the 2015 Zambia Living Conditions Monitoring Survey (LCMS) data, the 2017 Zambia NCD STEPS Survey, and key parameters from the literature, we simulated the effect of a 25% SSB tax in Zambia on energy intake and the corresponding change in body mass index (BMI), obesity prevalence, deaths averted, life years gained and revenues generated using a mathematical model developed using Microsoft Excel. We conducted Monte Carlo simulations to construct 95% confidence bands and sensitivity analyses to account for uncertainties in key parameters. We found that a 25% SSB would avert 2526 deaths, though these results were not statistically significant overall. However, when broken down by gender, the tax was found to significantly avert 1133 deaths in women (95% CI 353 to 1970). The tax was found to potentially generate an additional US$5.46 million (95% CI 4.66 to 6.14) in revenue annually. We conclude that an SSB tax in Zambia has the potential to significantly decrease the amount of disability-adjusted life years lost to lifestyle-related diseases in women, highlighting important health equity outcomes. Women have higher baseline BMI and therefore are at higher risk for NCDs. In addition, an SSB tax will provide government with additional revenue which if earmarked for health could contribute to healthcare financing in Zambia.

**Key questions**

**What is already known?**

- Sugar taxes could reduce the consumption of sugary beverages, which in turn could improve population health and generate revenues in middle-income and high-income countries. However, there is limited or no evidence in low-income and middle-income countries (LMICs).

**What are the new findings?**

- A sugar tax in an LMIC context like Zambia would also reduce the consumption of sugary drinks, generate additional resources and improve health outcomes.
- Improvements in health outcomes are stronger in women.

**What do the new findings imply?**

- A sugar tax may have strong equity effects by improving health more among women, who are more likely to have higher obesity-related problems due to higher body mass index.

## Background

Non-communicable diseases (NCDs) are a leading cause of death and disability, accounting for over half of all deaths worldwide in 2015.^1^ Central to the increasing burden of NCDs is the rise in the prevalence of known risk factors such as obesity.^2 3^ Obesity prevalence has been rising at an alarming rate, increasing threefold since 1975 with current statistics showing that approximately 13% of adults are obese.^4^ Recently, the rise in the prevalence of obesity has been much higher in sub-Saharan Africa; increasing from 5% to 15% between 2000 and 2016.^5^

The rapid rise in global obesity rates has been attributed to a number of lifestyle factors, among them the excessive consumption of sugary foods.^6^ A number of studies have demonstrated that increased sugar intake is associated with increases in obesity^6–8^ and diabetes.^9^ One major concern is that consumption of sugar sweetened beverages (SSBs) has been increasing globally, rising by 5.6% for men and 9.2% for women between 1990 and 2015.^10^ A similar trend has been observed in low-income and middle-income countries (LMICs),^11^ where at least three in every five adolescents now consume SSBs daily compared with two in every five adolescents in high-income countries.^12^

Like many LMICs, the burden of NCDs in Zambia has been growing. Between 2009 and 2011, the burden of NCDs in Zambia increased by over 50%.^13^ By 2016, close to a quarter of all deaths in the country were attributed to NCDs.^14^ The Zambia Sample Vital Registration with Verbal Autopsy 2015/2016 report indicated that more than a third of NCD-related deaths were linked to alcohol consumption whereas 20.4% were linked to tobacco smoking.^15^ NCD deaths due to diseases of the circulatory system were the most common at 55.3% of all NCD deaths, followed by cancers at 14.8% whereas endocrine, nutrition and metabolic-related diseases accounted for 1 in 10 of all NCD deaths.^15^

The rising obesity prevalence is a growing problem, especially among women. Between 2001 and 2014, the number of overweight and obese women in Zambia increased by about 75%.^16^ As of 2014, approximately one in five adult women in Zambia were either obese or overweight.^16^ At the same time, consumption of SSBs has been increasing steadily. Specifically, consumption of SSBs produced by Zambia Breweries—the largest distributor of soft drinks—grew by about 4% in just 1 year—between 2015 and 2016.^17^ The increase in consumption might have been driven by increases in average income levels given Zambia’s real gross domestic product (GDP) per capita increased by 1% between 2015 and 2016.^18^ The increased consumption of SSBs during this period was also driven by a reduction in average SSB retail prices by major producers.^17^

The increased disease burden associated with increasing sugar intake could exacerbate the strain on already fragile health systems in LMICs in sub-Saharan Africa, which require increasingly more resources to manage the increase in the burden of NCDs. Most sub-Saharan African countries have limited revenue-generating capacity, relying on external financing for about 40% of their total health expenditure.^19^ Against this backdrop researchers, scientists and policy makers have called for action to curb the consumption of SSBs.^20^ One of the most promising options to do this is to impose a tax on SSBs. Such a tax has the potential to reduce consumption of SSBs, improve health and increase tax revenue at the same time.^21^

A rich literature simulating the likely effect of an SSB tax (also known as a sin tax) has shown that it could reduce consumption of SSBs, reduce obesity, lower diabetes and avert a substantial number of deaths.^22–26^ Similar evidence on consumption effects is found in observational evaluations in countries that have implemented the tax.^3 27^ It has also been shown that such a tax would increase revenue, which could be used for health promotion programmes.^21^ Furthermore, NCDs may reduce worker productivity as well as overall household incomes.^28^ Thus, taxing SSBs could boost economic productivity and households’ earnings and savings.^29^

However, there is a complex political economy surrounding the taxation of SSBs. As already noted, public health promoters propose that a ‘sin’ tax on substances such as SSBs, alcohol and tobacco would have significant health benefits. This is a tax on goods that negatively affects physical and mental health. For SSB-related firms, such a tax may lead to job losses due a reduction in the demand for SSBs.^30^ For political actors, while the tax would potentially increase tax revenues,^31^ they also worry that it raises critical equity concerns as it may disproportionately affect the poor.^32^ Wilde *et al* found that an SSB tax would be cost-saving from a governmental, health sector and societal viewpoint, and increase revenues for the government but would be costly for the beverages industry.^33^ Therefore, introducing the tax would require a tight balancing act of the interests of various stakeholders.^34 35^

Although the literature on SSB taxation is rich, it has mainly focused on high-income and middle-income countries. Notable studies include Colchero *et al* on Mexico, which found that a 10% tax on sugary drinks reduced their consumption by 12% and obesity prevalence by 3.8% in men and 2.4% in women;^27^ and Falbe *et al* on Berkeley in the USA which found that $0.01/oz tax on sugary drinks reduced their consumption by 21%.^36^ There is limited evidence on the likely effects of an SSB tax in LMICs in sub-Saharan Africa. Notable exceptions are Manyema *et al* and Stacey *et al*,^37 38^ which used mathematical models to simulate the impact of an excise tax on SSBs in South Africa. These and similar studies have been influential in advancing the academic and policy debate for the use of ‘sin’ taxes in South Africa; which implemented a 2.1 cents per gram of sugar (above the 4 grams of sugar per 100 mL threshold) tax on sugary beverages in April 2018. The aim of this study is to model the impact of a 25% sugar tax on consumption of SSBs, health benefits and revenue generation in Zambia, an LMIC.

## Methods

We considered the impact of 25% excise tax on SSBs. The optimal tax rate was arrived at through consultations at a validation meeting with officials from the Ministries of Health, Finance, and National Development Planning and with other stakeholders. After simulations on different tax rates, stakeholders were of the view that a 25% tax would maximise direct and indirect health and revenue benefits. The Zambian government levies excise duty on other commodities such as alcohol and cigarettes ranging from 40% for clear beer to 145% for tobacco.^39^ Therefore a 25% tax on SSBs falls well within the limits of prevailing excise taxes on other commodities.

### Data sources

Data and parameters of interest for this study were obtained from multiple sources as depicted in table 1.

**Table 1:** Data sources

Parameter/variable	Best estimate	Source
BMI (kg/m^2^)	23.38	Zambia STEPS Survey 2017
Average weekly consumption of sugary drinks (300 mL servings)	1.55	Zambia Living Conditions and Monitoring Survey (LCMS) 2015
Average price of sugary drinks (per 300 mL serving)	ZMW 3.77 (or US$0.40)	Zambia Central Statistics Office
Average all-cause mortality rate (per 100 000 population)	30.1	Zambia Central Statistics Office
Potential impact fractions (PIF) (1+ PIF)	0.0001 (1.0001)	Manyema *et al*^38^
Own-price elasticities for SSBs	−1.30 (95% CI −1.10 to –1.51)	Meta-analysis
Cross-price elasticities	0.32 (95% CI 0.01 to 0.77) for fruit juice, and 0.18 (95% CI −0.10 to 0.34) for milk.	Escobar *et al*^45^
**Pass-on rate**	100% (80%–120%)	Crawford *et al*,^41^ Berardi, Sevestre *et al*,^42^ and Manyema *et al*^38^

BMI, body mass index; CI, confidence interval; SSB, sugar sweetened beverage; STEPS, STEPwise approach to noncommunicable disease risk factor surveillance; ZMW, Zambian Kwacha.

Consumption data were obtained from the 2015 Living Conditions Monitoring Survey (LCMS) data set. The LCMS is the largest and richest nationally representative survey that collects information on household conditions including housing, consumption and income. Average weekly SSB consumption was 1.55 of 300 mL servings (see table 1).

Information on beverage consumption was collected by asking households about the quantity of beverages they consumed in the 2 weeks preceding the survey. However, this study focused only on beverage consumption among the adult population. Thus, the study population was all adult members of the overall Zambian population.

To adapt the LCMS data set for use, we standardised all reported consumption amounts into litres. Next, the household consumption of the various beverages were translated into equivalent 300 mL servings. The data were reported in terms of fortnightly consumption. The observed 300 mL servings were divided by two to give the weekly beverage consumption; and then by corresponding household sizes to obtain weekly per capita consumption. The last step in the (consumption) data analysis was the allocation of per capita beverage consumption to different age and sex groups based on allocation shares from work by Manyema *et al;*^38^ which used data from the 2012 South African National Health and Nutrition Examination Survey.

The baseline population used in the life table was based on the 2011 census population projections report.^40^ The total projected population in 2015 was 15 473 905. The adult population considered in the study (the population aged 15 years and above) numbered 8344 486, of which 51% (4271 631) was female and 49% (4 072 855) was male. Age-specific all-cause mortality rates were obtained from the Central Statistics Office (CSO). The average all-cause mortality rate was 30.1 per 100 000 population. A discount rate of 6% was used to find discounted life years under both the baselines and outcome scenarios. The life table was further stratified by sex allowing us to estimate health outcomes for men and women separately.

Data on average price of beverages were obtained from the price department of the Zambia CSO. Fruit juice was the most expensive of three beverages at an average price of ZMW4.93 or US$0.52 per 300 mL serving. This was followed by SSBs, which cost roughly ZMW3.77 or US$0.40 per 300 mL servings (see table 1). The average price of milk was ZMW3.64 or US$0.38 per 300 mL serving (the 2017 average ZMW/US exchange rate of 9.53 was used).

To compute the body mass index (BMI), data on weight (in kilograms) and height (in metres) for various age groups were derived from the 2017 STEPS Survey data set. The traditional BMI formula, weight/height^2^ was used, giving the standard unit of measurements, kg/m^2^. The average BMI was 23.38 kg/m^2^ (see table 1).

### The model

We used a mathematical model to simulate the impact of the tax on three main outcomes, namely, SSB consumption, DA and revenue generation, over a 40-year time horizon.^37^ The 40-year time horizon was chosen because it more reasonably tracked health outcomes over the life cycle of the adult population (cohort) that was alive at the time of the simulation. The impact of the tax was computed as the difference in consumption, number of deaths, and tax revenues between their realisation in an intervention scenario (with a 25% excise tax in place) and the baseline scenario (without an excise tax). All health outcomes reported—life years gained (LYG) and deaths averted (DA)—are the total outcomes for the entire 40-year time horizon, while revenue outcomes were annualised.

The analysis involved four main steps, describing how a tax affects key outcomes. In step 1, a 25% excise tax increases the price of SSBs. The effect on the price depends on the parameter *pass-on rate*—the proportion of the tax that is passed on to consumers in the form of higher retail prices. We assumed a 100% *pass-on rate* in line with Crawford *et al* and Berardi *et al*.^41 42^ However, some studies find a pass-on rate of less than 100%,^43^ while others find a pass-on rate of more than 100%.^44^ A pass-on rate of 100% is a conservative estimate and has been applied in similar empirical contexts such as South Africa.^38^

Second, we examined how the increased price affects consumption of SSBs. The strength of this effect depends on two key parameters; (1) The *own-price elasticity*, which is the responsiveness of changes in consumption of SSBs to changes in their price. (2) The *cross-price elasticity,* which shows the strength of the substitution away from SSBs to other now relatively cheap substitutes. The substitutes considered were milk and fruit juice. *Own-price elasticities* were based on estimates of −1.37 and −1.18 from previous studies.^37 38^ Our study used a conservative own-price elasticity of demand of −1.30 based on a comprehensive meta-analysis, a reasonable estimate not significantly different from recent estimates from South Africa.^37^ C*ross-price elasticities* of 0.32 for fruit juice and 0.18 for milk were based on a previous study.^45^ Baseline consumption data for milk, juice and soft drinks were obtained from the 2015 LCMS.

Third, we examined how changes in consumption of SSBs and their substitutes affects energy intake. The impact on energy intake may be high or low depending on consumers’ incentives to substitute taxed SSBs for other high-energy beverages such as fruit juice and milk. Changes in SSB energy intake reduced daily energy intake proportionately, leading to a shift in BMI distribution of the population, and hence obesity prevalence. Data on the baseline BMI distribution were obtained from the 2017 Zambia STEPS Survey. Our measurement of the extent to which changes in daily energy intake affect weight/BMI is based on Swinburn *et al*, where a daily energy intake of 94 KJ/day over and above the daily recommended allowance is associated with a 1 kg increase in the lifetime weight of an average adult.^46^

**Figure 1:**
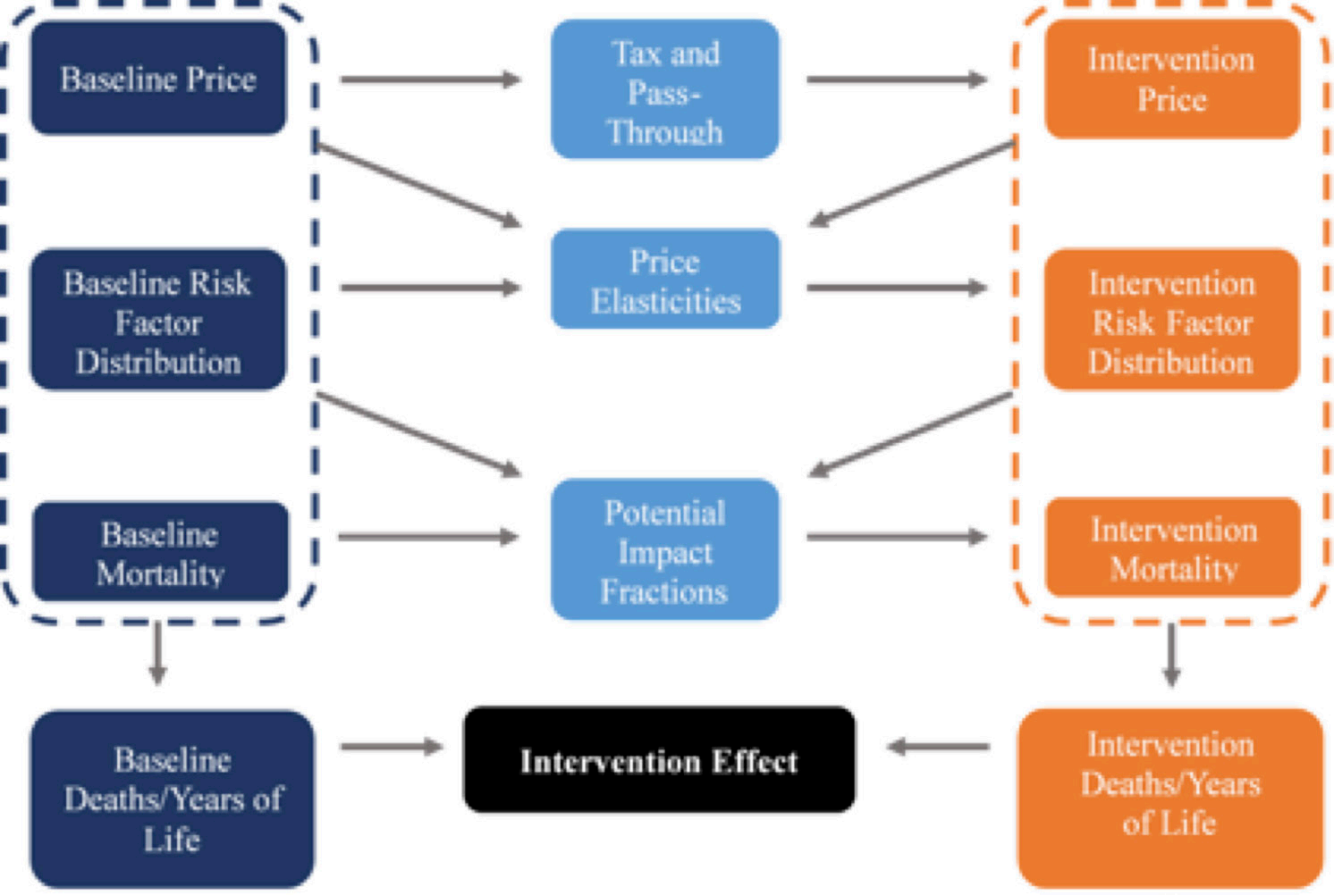
Model simulation structure.

Finally, once the impact of energy intake on BMI distribution is computed, the impact of changes in BMI on mortality is estimated. The Zambia life tables were obtained from CSO. From the baseline life tables, the number of years lived and number of people dying is computed using potential impact fractions (PIFs). Our PIFs measure the percentage change in the risk of a death resulting from a change in BMI. They are based on risk ratios linking BMI and mortality. Average relative risks were 1.16 for a BMI less than 18.5 kg/m^2^, 1.03 for a BMI of between 18.5 kg/m^2^ and 20.9 kg/m^2^, 1.00 for a BMI between 21 kg/m^2^ and 22.9 kg/m^2^, 1.00 for a BMI between 23.0 kg/m^2^ and 24.9 kg/m^2^, 1.06 for a BMI between 25.0 kg/m^2^ and 26.9 kg/m^2^, 1.13 for a BMI between 27.0 kg/m^2^ and 29.9 kg/m^2^, 1.13 for a BMI between 30.0 kg/m^2^ and 34.9 kg/m^2^, and 1.59 for a BMI greater than 35 kg/m^2^.^47^ These were further disaggregated by sex. Comparison on the years of life lived and number of people dying in the intervention and baseline Markov life table gives LYG and DA. The model simulation structure is summarised in figure 1 below.

An uncertainty analysis using Monte Carlo simulation with Visual Basic in Microsoft Excel 2013 was undertaken to account for uncertainty arising from statistical variation in model parameter estimates. The four steps simulated were repeated 500 times. In each simulation, all the parameters are drawn from an inverse normal distribution whose means are the parameter estimates described above. The impact of the tax, for example on life years, is computed by averaging the LYG in all the 500 simulations. The 5th and 95th percentiles of these values formed the confidence bands.

The impact of the tax on revenue is similarly calculated. The change in tax revenue took into account the percentage of the Zambian population consuming SSBs (13%) based on the 2015 LCMS.^48^ Changes in total revenue due are derived from the second step, which is the change in consumption of SSBs. A reduction in SSB consumption could lead to a fall in value added tax (VAT) revenue but increase excise tax revenue. The revenue effects of the tax are calculated as the difference between the tax revenue generated under the simulation and baseline scenarios. The revenue effect is therefore the sum of the changes in the excise revenue from the SSB tax and changes in VAT collected/lost on SSB and on related beverages such as juice and milk.

### Sensitivity analyses

Given the uncertainty in key parameters, we used sensitivity analyses to determine how changing one or more parameters affects the results of the simulation.^49^ We distinguished between (one-way and two-way) deterministic and probabilistic sensitivity analyses. In the deterministic sensitivity analysis, we analysed the impact of varying the tax and pass-on rates on the health and revenue effects of the sugar tax. The tax rate was varied between 15% and 25% while the pass-on rate was varied between 80% and 100%. Following Briggs *et al*, these parameters were targeted given their uncertainty and lack of country-specific evidence of their values.^22^

The study also used probabilistic sensitivity analysis to allow for the variation of all key parameters simultaneously. Probabilistic sensitivity analysis allowed us to assign an appropriate distribution for each key parameter and vary them probabilistically.^50^ The parameters subjected to probabilistic sensitivity analysis include the own-price and cross-price elasticities, and the relative risks of BMI-related mortality.

### Patient and public involvement

Given the nature of the research, there was no involvement of patients.

### Ethics

This study relied on secondary data from the 2015 LCMS and 2017 STEPS Survey. The data set was anonymised.

## Results

### Baseline consumption

About 12.8% of households consumed SSBs in 2015 compared with 12.0% in 2010 in the 2 weeks preceding the survey. Per capita household consumption was allocated to different age and sex groups using allocation shares from work by Manyema *et al*.^38^ Table 2 summarises the baseline per capita weekly beverages consumption by each beverage type.

**Table 2:** Weekly per capita beverage consumption by sex and age groups

Age, years	Average weekly consumption of 300 mL servings		
	SSB	Fruit juice	Milk
Male			
15–24	1.77	1.47	0.98
25–34	2.17	1.32	1.21
35–44	2.05	1.30	1.31
45–54	1.82	1.12	1.28
55–64	1.52	1.11	1.27
65+	1.21	1.22	1.30
Female			
15–24	1.71	0.79	1.01
25–34	1.73	1.52	1.15
35–44	1.70	1.52	1.31
45–54	1.61	1.46	1.29
55–64	1.30	1.30	1.21
65+	0.93	1.20	1.25

SSB, sugar sweetened beverage.

From table 2, consumption of SSBs is highest in the 25–34 years age group for both men and women. Furthermore, SSB consumption decreases as age increases for both men and women.

### Effects on SSB consumption

**Figure 2:**
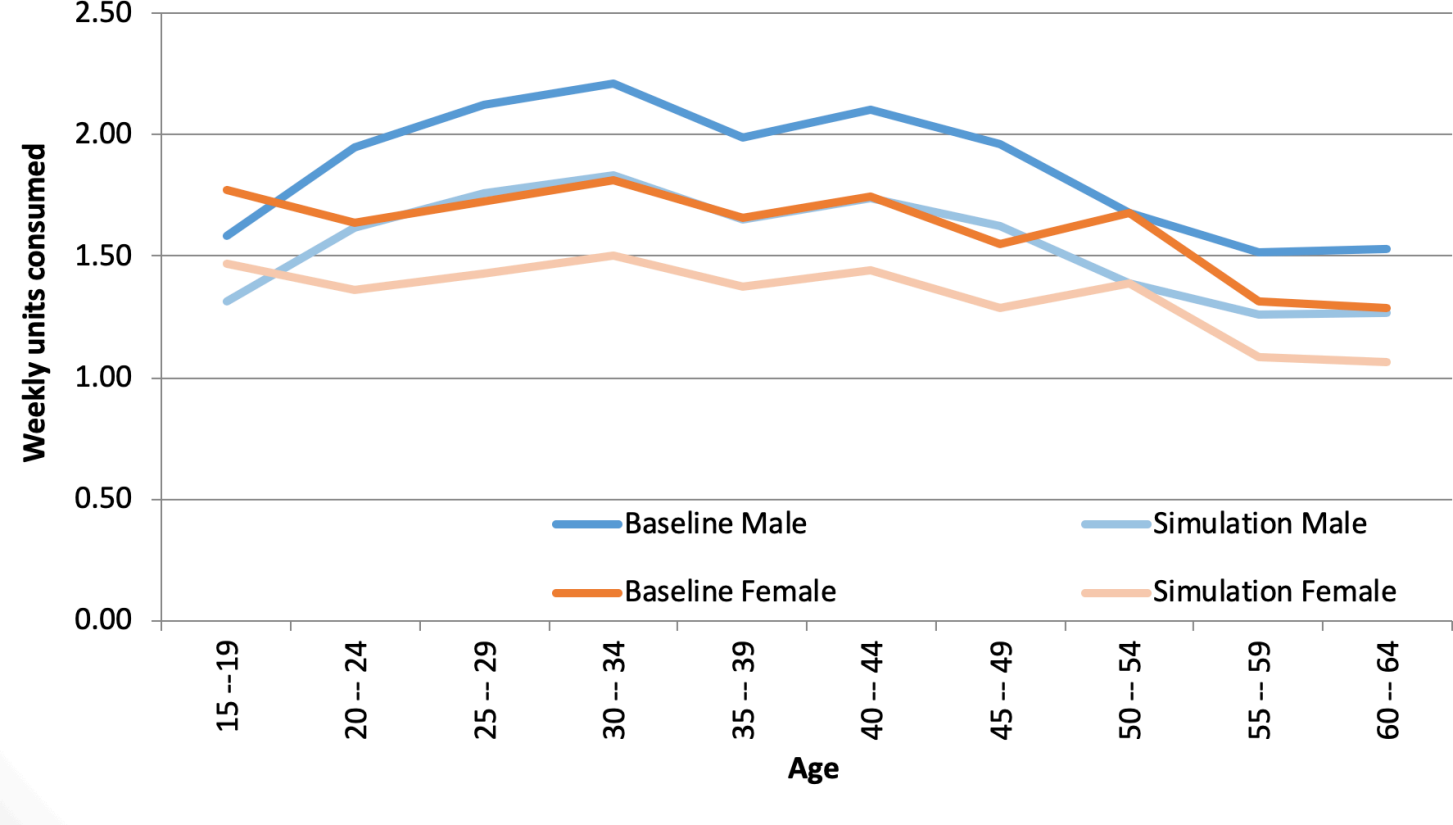
Impact on consumption of sugary drinks.

Our results show that a 25% tax increased the retail price of SSBs by approximately ZMW0.94 (or US$10). This increase was expected to reduce weekly SSB consumption by approximately 0.56 units of 300 mL servings, on average (95% CI 0.51 to 0.61). Figure 2 shows baseline and simulated impact of the tax on weekly consumption across age groups.

### Effects on energy intake, body mass and obesity prevalence

The reduction in consumption of SSBs has implications for energy intake, BMI and obesity prevalence. The reduction in consumption was associated with a reduction in the average daily energy intake of 33.0 KJ (95% CI 29.2 KJ to 36.8 KJ).

The reduction in energy intake was associated with an average reduction in the BMI of 0.13 kg/m^2^ (95% CI 0.12 to 0.14). The reduction in BMI was similar in both sexes even though the average baseline BMI was greater in women (24.3 kg/m^2^ (95% CI 23.84 to 24.69)) compared with men (22.5 kg/m^2^ (95% CI 22.18 to 22.81)).

**Figure 3:**
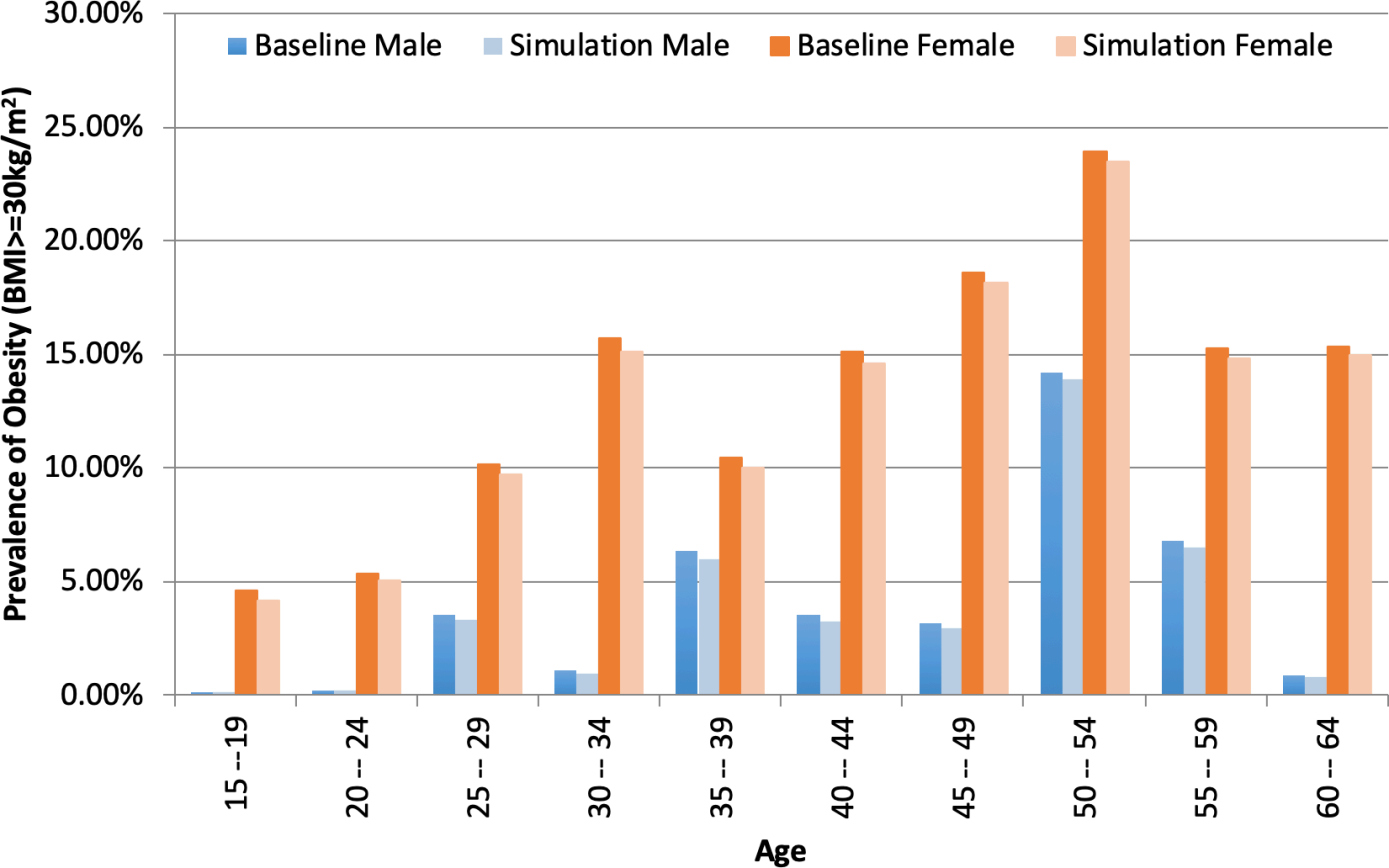
Effect of a 25% sugar tax on obesity prevalence. BMI, body mass index.

Obesity prevalence reduced by 0.49 percentage points (95% CI 0.41 to 0.57). The reduction was significantly higher in women than in men. Figure 3 below shows that the average baseline obesity prevalence was higher for women (13.5% (95% CI 9.24% to 17.69%)) than men (3.99% (95% CI 0.92% to 7.06%)). Baseline obesity prevalence was highest in the 45–49 years and 50–54 years age groups for both men and women.

### Effects on health outcomes

All the health outcomes are based on simulation over a 40-year period. The tax led to 14 755 LYG (95% CI −11 965 to 42 701) and 2526 DA (95% CI −1743 to 6959), although on average these effects were not statistically significant (table 3). However, breaking down the effect by sex reveals the tax has a significant effect on women. The tax would significantly lead to between 2006 and 11 910 LYG, and 353 and 1970 DA.

**Table 3:** Deaths averted and life years gained

	Mean	95% lower bound	95% upper bound
Deaths averted			
Male	1 to 393	−2 to 912	5 to 694
Female	1 to 133	353	1 to 970
Total	**2 to 526**	−**1 to 743**	**6 to 959**
Life years gained			
Male	7 to 930	−19 to 366	34 to 786
Female	6 to 824	2 to 006	11 to 910
Total	**14 to 755**	−**11 to 965**	**42 to 701**

Given the uncertainties in the tax and pass-on rates, we conducted one-way sensitivity analysis to determine how varying these parameters affect the health benefits. However, a key limitation of this deterministic sensitivity analysis is that it was not extended to other uncertain parameters or variables such as PIFs, the all-cause mortality rate and average SSB prices, all of which did not have reliable CIs or SEs. We found that increasing the tax rate led to increased health benefits. A tax rate of 15% was associated with an average of 7731 LYG and 1335 DA compared with 10 041 LYG and 1549 DA for a 20% tax rate, and 14 755 LYG and 2526 DA for a 25% tax (assuming a 100% pass-on rate).

Similarly, health effects are positively correlated with the pass-on rate. That is, a higher pass-on rate is associated with higher health benefits and vice versa. For example, an 80% pass-on rate was on average associated with 9921 LYG and 1722 DA compared with 14 755 LYG and 2526 DA for a 100% pass-on rate (assuming a 25% tax rate).

A two-way sensitivity analysis was conducted to assess how varying the two parameters simultaneously would impact health outcomes. As expected, it was found that LYG and DA are highest if both the tax and pass-on rate are high. For example, a tax rate–pass-on rate combination of (15%, 80%) was on average associated with 6345 LYG and 1100 DA compared with 14 755 LYG and 2526 DA for a (25%, 100%) combination (see table 4).

**Table 4:** Effect of varying tax and pass-on rates on health effects

	Tax rate			
		15%	20%	25%
**Life years gained**				
Pass-on rate	80%	6 to 345	8 to 777	9 to 921
	90%	7 to 339	9 to 514	12 to 415
	100%	7 to 731	9 to 921	14 to 755
**Deaths averted**				
Pass-on rate	80%	1 to 100	1 to 510	1 to 722
	90%	1 to 262	1 to 642	2 to 133
	100%	1 to 335	1 to 722	2 to 526

### Effects on revenue generation

As noted earlier, a sugar tax can also be used to generate resources to supplement the health budget and to mobilise resources for public health programmes.

Overall, the tax has potential to raise about US$ 5.46 million annually (the Bank of Zambia 2017 average ZMW/US exchange rate of 9.53 was used (see: https://www.boz.zm/average-exchange-rates.htm for historic ZMW/US$ exchange rates) (95% CI 4.66 million to 6.14 million) (table 5). The additional revenue generation would largely be the result of an increase in the SSB excise tax revenue. However, there is a slight reduction in VAT revenue. To put this into context, the projected average annual revenue from the SSB tax would be equivalent to about 1% of the total national budgetary allocation towards the health sector (US$633 million) and about 8% (or US$70.3 million) of the budgetary allocation for drugs and medical supplies in 2019.^51^

**Table 5:** Annual tax revenue generated, US$ million

	Mean	95% lower bound	95% upper bound
Excise revenue	5.99	5.58	6.35
VAT revenue	−0.53	−0.92	−0.14
Total	5.46	4.66	6.14

N/A, N/A; VAT, value added tax.

Given uncertainties about the tax rate that may be adopted—often the result of a complex political economy process—we conducted one-way sensitivity analysis to assess the effect of varying the tax rate on revenue generated. Again, a key limitation of this analysis is its inability to adequately account for uncertainties in PIFs, the all-cause mortality rate and average SSB prices. As expected, a higher tax rate was associated with a higher revenue generated. For example, a 20% sugar tax would raise about US$4.91 million (95% CI 4.38 to 5.43) annually compared with US$5.46 million (95% CI 4.66 to 6.14) for a 25% tax (assuming a 100% pass-on rate). The pass-on rate has a similar effect. An 80% pass-on rate would on average raise US$4.91 million annually (95% CI 4.38 to 5.43) compared with US$ 5.46 million (95% CI 4.66 to 6.14) for a 100% pass-on rate (assuming a 25% tax rate).

The two-way sensitivity analysis showed that revenue effects are highest when the tax and pass-on rates are relatively high. A tax–pass-on rate combination of (15%, 80%) would raise US$3.44 million annually compared with US$5.46 million for a (25%, 100%) combination. In practice, revenue generated would be somewhere in between the two values; given that the actual tax rate would be a product of a complex political economy process, and the degree of pass-on is an empirical question.

## Discussion

This study analysed the impact of a 25% tax on SSBs on consumption, health and revenue outcomes. The study has shown that the introduction of this tax has the potential to reduce consumption of SSBs and lead to health benefits, particularly among the female adult population.

The reduction in SSB consumption and obesity prevalence was similar to what has been found in other empirical studies.^3 27^ However, our study found that the reduction in obesity prevalence was greater among women than men. This is largely at odds with the findings of other empirical studies which found that the reduction in obesity prevalence was greater among men compared with women.^23 25 27^ The greater impact of an SSB excise tax on obesity prevalence among women observed in the present study may be explained by the relatively greater baseline obesity prevalence and PIFs among adult women compared with adult men in Zambia. This raises important equity considerations. Given well-established evidence that women are likely to record worse health outcomes and hence use healthcare services more often,^52^ Zambian women could be the biggest health beneficiaries of an SSB tax. This has the potential to reduce gender inequities in heath.

In terms of revenues, the impact of a sugar tax appears to be substantially lower in Zambia than what was found in other countries.^38^ This could be because the total population and proportion of the population that consume SSBs is much lower in Zambia. Nonetheless, this additional revenue could be earmarked for public health programmes that promote healthier diets and lifestyles. Furthermore, these extra revenues could also be used to supplement the newly introduced National Health Insurance (NHI) fund.

A major limitation of our study is the inability to incorporate the effect of changes in wider variables such as per capita income, proportion of households consuming SSBs and beverages inflation on the impact of a sugar tax. Changes in key macroeconomic variables have the potential to amplify or dampen the consumption, health and revenue effects of a sugar tax over time. For example, beverages inflation, especially if the SSB tax is lump sum, could imply that the size of the tax becomes insignificant as a percentage of the retail price.

Another limitation of the study is its inability to account for seemingly ‘irrational’ behavioural and psychological responses—at least as far as standard economic theory is concerned—that may underlie economic agents’ response to a sugar tax. Such behavioural and psychological responses could potentially dampen the effects of a sugar tax.^53^ In addition, the study did not take into account the effect of changing consumer preferences over time; although it accounted for ‘static’ substitution possibilities across different types of beverages.

Lastly, another significant limitation of the study is that the study relied on meta-analyses for estimates of key parameters such as price elasticities and pass-on rates owing to the absence of Zambia-specific estimates. This could have major implications on the findings since the meta-analyses are based on data from countries whose economic characteristics may somewhat differ from that of Zambia. Nevertheless, the study mitigated against uncertainties involving key parameters by employing deterministic and probabilistic sensitivity analyses.

## Conclusion

This study investigated the impact of a 25% tax on SSBs on their consumption and corresponding health and revenue effects in Zambia. We showed that such a tax has the potential to reduce the consumption of SSBs and may have a positive impact on LYG and revenue generated.

The health benefits were significant among women, possibly due to higher baseline BMI and obesity prevalence and PIFs among women. Thus, women are likely to be the biggest beneficiaries of a tax on SSBs. The implication of this finding for dealing with the NCD burden is that one-size-fits-all interventions that are gender-insensitive are likely to lead to suboptimal outcomes.

The tax has the potential to raise up to US$5.46 million in revenue annually. These additional revenues could be used to supplement public health promotion programmes that encourage healthier lifestyles. Furthermore, the country’s health system is at a critical juncture with the introduction of an NHI scheme. Extra revenues generated could supplement the scheme.

Given that the magnitude of the effects of the tax may be influenced by long-term changes in key macroeconomic fundamentals such as per capita income and inflation, there must be a mechanism for periodic review of the size of the tax as key macroeconomic fundamental variables and consumer tastes and preferences change over time.

In conclusion, this paper contributes to the wider literature on an evidence-based priority setting for the improvement of health of the Zambian population. In particular, it is hoped that it lays the ground for further work on evidence-based policy recommendations on ‘sin’ taxes in Zambia, and their implications for population health.
